# A Combined in Silico and Structural Study Opens New Perspectives on Aliphatic Sulfonamides, a Still Poorly Investigated Class of CA Inhibitors

**DOI:** 10.3390/biology12020281

**Published:** 2023-02-10

**Authors:** Emma Langella, Davide Esposito, Simona Maria Monti, Claudiu T. Supuran, Giuseppina De Simone, Vincenzo Alterio

**Affiliations:** 1 Institute of Biostructures and Bioimaging-CNR, Via Pietro Castellino 111, 80131 Naples, Italy; 2 Neurofarba Department, Section of Pharmaceutical and Nutriceutical Sciences, Università degli Studi di Firenze, Via Ugo Schiff 6, 50019 Sesto Fiorentino, Italy

**Keywords:** carbonic anhydrase inhibitors, aliphatic sulfonamide, X-ray crystallography, binding free energy calculations, structure-based drug design

## Abstract

**Simple Summary:**

Carbonic anhydrases are a family of enzymes that catalyze an essential physiological reaction for living organisms: the reversible conversion of CO_2_ to bicarbonate ion. In humans, these enzymes impact many physiological and pathological processes including respiration, pH and CO_2_ homeostasis, electrolyte secretion, gluconeogenesis, ureagenesis, lipogenesis, bone resorption, and tumorigenicity. For this reason, several human carbonic anhydrases have become therapeutic targets for the treatment of many disorders. In recent years, a huge number of carbonic anhydrase inhibitors have been developed for therapeutics aims, such as diuretic, antiglaucoma, antiobesity, and anticonvulsant agents, and for the diagnosis and treatment of cancer diseases. The authors report a combined crystallographic and computational study on a promising class of carbonic anhydrase inhibitors to clarify their mechanism of action and to obtain useful information for the drug design of new effective and selective molecules.

**Abstract:**

Aliphatic sulfonamides are an interesting class of carbonic anhydrase inhibitors (CAIs) proven to be effective for several carbonic anhydrase (CA) isoforms involved in pathologic states. Here we report the crystallographic structures of hCA II in complex with two aliphatic sulfonamides incorporating coumarin rings, which showed a good inhibition and selectivity for this isoform. Although these two molecules have a very similar chemical structure, differing only in the substitution of the two aliphatic hydrogen atoms with two fluorine atoms, they adopt a significantly different binding mode within the enzyme active site. Theoretical binding free energy calculations, performed to rationalize these data, showed that a delicate balance of electrostatic and steric effects modulate the protein-ligand interactions. Data presented here can be fruitfully used for the rational design of novel and effective isozyme-specific inhibitor molecules.

## 1. Introduction

Carbonic anhydrases (CAs; EC 4.2.1.1) are widespread metalloenzymes which catalyze the reversible hydration of carbon dioxide (CO_2_ + H_2_O ⇆ HCO_3_^−^ +H^+^) [1]. Eight distinct genetic families, namely α-, β-, γ-, δ-, ζ-, η-, θ-, and ι-CAs, have been so far identified in the different living organisms [1,2,3,4,5,6,7,8,9]. In particular, CAs belonging to the α-family have been found in fungi, vertebrates, corals, protozoa, algae, bacteria, and green plants. β-CAs are highly distributed in plants but members of this class have also been found in bacteria, fungi, algae, and archaea. Members of the γ-class have been found in plants, archaea and bacteria, δ- and ζ-CAs have been identified in marine diatoms, whereas three new isoforms were recently identified in diatoms, bacteria, algae, and archaea (ι-CAs), in the pathogenic protozoan *Plasmodium falciparum* (η-CA), and in the diatom *Phaeodactylum ricornutum* (θ-CAs) [2,4,5,6,7,8]. Despite the different three-dimensional structures characterizing the eight classes of CAs, with the exception of the iota one [10] all contain a divalent metal ion in their active site, essential for the catalytic activity. In almost all CAs this is a Zn(II) ion, even if γ-CAs probably contain a Fe(II) in the active site whereas the CAs belonging to the ζ-class are cambialistic enzymes, with enzymatic activity with both Cd or Zn ions [11].

All human CAs (hCAs) belong to the α-family, which is the most populous, with 15 isoforms differing for molecular features, kinetic properties, oligomeric arrangement, and cellular localization [12,13]. In detail, eight isoforms are localized in the cytosol (CAs I, II, III, VII, VIII, X, XI and XIII), four are associated to the cell membrane (CAs IV, IX, XII and XIV), two are confined in mitochondria (CAs VA and VB), and one is secreted in milk and saliva (CA VI) [1]. hCA isoforms also differ in their enzymatic efficiency, with some of them being among the most active enzymes currently known (isoforms II, VB, VII, and IX), while others (isoforms VIII, X, and XI) are devoid of any enzymatic activity [1,14].

A huge number of structural studies have been carried out on α-CAs, showing that, in agreement with their high sequence homology, all of these enzymes have a very similar three-dimensional structure, regardless of their oligomeric state or cellular localization. The main structural features are represented by a central twisted β-sheet enclosed by helical regions and additional β-strands [1,15]. The enzyme active site is positioned in a deep and large cavity, with the catalytic zinc ion placed on its bottom coordinated by three conserved histidine residues (His94, His96 and His119) and a water molecule/hydroxide ion which acts as nucleophile in the reversible hydration reaction of CO_2_ to bicarbonate ion [1,15]. 

The hydration reaction proceeds through a two-step mechanism. The first step consists of a nucleophilic attack by the zinc-bound hydroxide ion on the CO_2_ molecule, leading to the formation of HCO_3_^−^ which is subsequently displaced from the catalytic site by a water molecule (Equation (1)). Subsequently, in the second and rate limiting step, the zinc-bound hydroxide ion is regenerated by the transfer of a proton from the zinc-bound water molecule to the bulk solvent (B) (Equation (2)) [1,14].

EZn^2+^-OH^−^ + CO_2_ ⇆ EZn^2+^-HCO_3_^−^ ⇆ EZn^2+^-H_2_O + HCO_3_^−^ (1)

EZn^2+^-H_2_O + B ⇆ EZn^2+^-OH^−^ + BH^+^ (2)

The availability of the crystallographic structures of hCA isoforms allowed the identification of two very different environments within the enzyme active site cavity: one region delimited by hydrophobic amino acids and one mainly constituted by hydrophilic residues [1]. Several studies showed that the hydrophobic region is important for the capture of the CO_2_ substrate and its correct orientation in the active site in order to undergo the nucleophilic attack by the zinc-bound hydroxide ion. On the other end, the hydrophilic region enables the assembly of a well-ordered hydrogen-bonded solvent network that assists the transfer of the proton from the zinc-bound water molecule to the bulk solvent. The simultaneous presence of these two regions inside the active site allows the rapid catalytic cycling of CO_2_ to bicarbonate [1].

hCAs are extensively distributed in different tissues and organs, where they are involved in various physiological processes including pH and CO_2_ homeostasis, respiration, transport of CO_2_ and HCO_3_^−^, biosynthetic reactions such as lipogenesis, gluconeogenesis and ureagenesis, bone resorption, electrolyte secretion, calcification, and many others [16]. Consequently, their dysregulated expression and/or abnormal activity may have important pathological consequences [14,15]. For this reason, in the recent years these enzymes have been recognized by the scientific community as important targets for the design of inhibitors with biomedical applications [1]. Indeed, many CA inhibitors have been developed and some of them are used clinically, or in clinical trials, as antiglaucoma, diuretic, antiobesity, and anticonvulsant agents, and for the diagnosis and treatment of cancer diseases [15].

**Figure 1:**
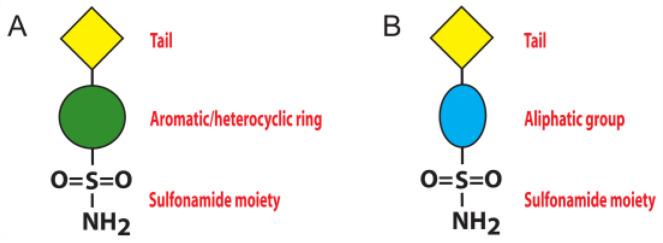
Schematic representation of sulfonamide CA inhibitors: (**A**) aromatic/heterocyclic sulfonamide; (**B**) aliphatic sulfonamide.

To date, the most investigated CA inhibitors (CAIs) are aromatic/heterocyclic sulfonamide derivatives (Figure 1A), which bind the enzyme with high affinity by coordinating in their deprotonated form the catalytic zinc ion with a tetrahedral geometry and establishing additional hydrophobic/polar interactions with residues delimiting the active site cavity [1].

**Figure 2:**
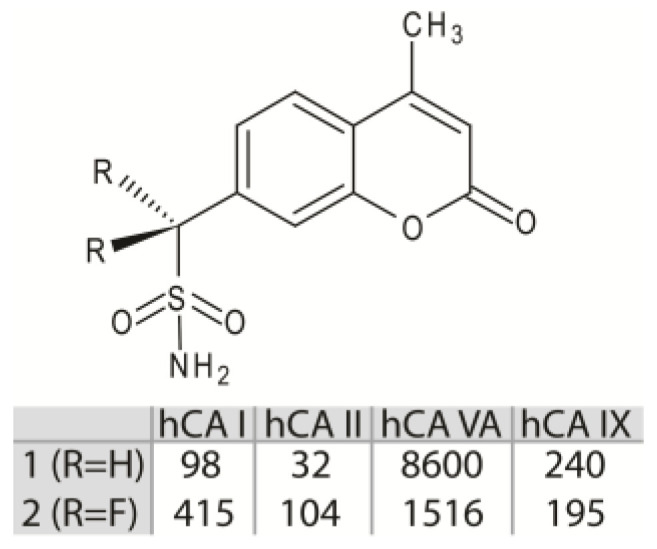
Chemical structures of compounds **1** and **2**. K_I_ value (nM) of these molecules against hCA I, II, VA and IX are reported [23].

However, since these molecules often lack selectivity for a specific CA isoform, their use as drugs for the treatment of CA related pathologies is strongly limited [1,15], and new inhibitor classes are continuously under investigation. Among these, the aliphatic sulfonamides (Figure 1B) have been scarcely studied, since for long time they were considered inactive as CAIs, principally due to the pKa of their solfonamide –NH_2_ group, generally higher with respect to that of the aromatic/heterocyclic sulfonamides [17,18]. This view was subsequently changed, since various aliphatic sulfonamides were shown to be potent inhibitors for several CA isoforms involved in pathologic states [19,20,21,22]. Thus, recently this class of compounds has begun to receive great attention from the scientific community, aimed at developing CAIs with improved selectivity and inhibition profiles compared to the classical aromatic and heterocyclic sulfonamides. In this context, in 2005 Cecchi and coworkers reported the synthesis of a library of substituted aliphatic sulfonamides incorporating phenyl, coumarin or steroidal rings and checked them for the inhibition of various hCA isoforms [23]. These compounds turned out to be potent inhibitors; in particular, the aliphatic sulfonamides **1** and **2** (Figure 2) containing a coumarin ring as tail, displayed very efficient inhibition of CA activity, with K_I_ values in the nanomolar range, and also showed a good selectivity for the isoform II [23]. 

Interestingly, from inhibition data analysis (Figure 2) it emerged that, although these two molecules possess a very similar chemical structure (they only differ for the substitution of the two aliphatic hydrogen atoms with two fluorine atoms), they showed a significant difference (more than three times) in the inhibition activity against the hCA II isoform [23]. Here, by high-resolution crystal structure of compounds **1** and **2** in complex with hCA II, together with theoretical binding free energy calculations, we elucidate the role of the molecular determinants responsible for the striking differences observed in the inhibition properties against this isoform.

## 2. Materials and Methods

### 2.1. Crystallization, Data Collection, and Structure Refinement

Compounds **1** and **2** were synthesized as reported by Taylor’s group [24], while hCA II protein was expressed and purified as previously described [25]. Crystals of the complexes between hCA II and compounds **1** and **2** were obtained by adding a 5-fold excess of each inhibitor to a 10 mg/mL protein solution in 20 mM Tris-HCl pH 8.0, 0.1% DMSO. 

The complexes were crystallized at 20 °C using a procedure previously described for other hCA II/inhibitor complexes [26]. In detail, crystals were obtained by the hanging-drop vapor diffusion method using 500 μL of reservoir solution containing 1.3 M Na-Citrate, 100 mM Tris-HCl pH 8.5. Crystallization drops were prepared by mixing 1 μL of complex solution with 1 μL of reservoir solution. Crystals appeared within three days and were used to collect complete datasets at 100 K, using a copper rotating anode generator developed by Rigaku and equipped with Rigaku Saturn CCD detector. Prior to data collection, crystals were briefly soaked in the crystallization buffer containing 20% (*v*/*v*) glycerol before being flash-cooled in liquid nitrogen. Diffracted intensities were processed using the HKL2000 program [27]. Data processing statistics are reported in Table 1. Structure analysis of the two complexes was done by difference Fourier techniques using as a starting model the crystallographic structure of hCA II crystallized in the P2_1_ space group (PDB code 4XE1) [28]. For both structures, a refinement protocol, consisting of an initial round of rigid body refinement followed by a slow-cool simulated annealing run at 2500 K, was used, in order to reduce possible model bias. In both cases, after a few rounds of refinement limited to the protein structure, a model of the inhibitor was inserted into the atomic coordinates set and further cycles of refinement were performed. Iterative rounds of manual model building (including side chains, water molecules, ligands and ions) and positional and individual B-value refinement were performed using the programs O [29] and CNS [30,31], respectively. Standard restraints for bond angles and distances were considered for protein atoms, while inhibitor distances and bond angle restraints were taken from the Cambridge Structural Database [32]. Water molecules included in the final model were built into peaks >3σ in |F_o_| − |F_c_| maps after checking their hydrogen-bonding geometry. Statistics for refinement are reported in Table 1. Coordinates and structure factors have been deposited with the Protein Data Bank (accession codes: 8C0Q, 8C0R).

### 2.2. Computational Study

Theoretical calculations were performed on the hCA II/**2** crystallographic complex and on the three model adducts hCA II/**2_ZBG_**, hCA II/2*_ZBG,_ and hCA II/2*_ring_. The model hCA II/**2_ZBG_** is identical to the corresponding crystallographic complex hCA II/**2** with the coumarin ring substituted with a hydrogen atom, whereas hCA II/2*_ZBG_ is identical to hCA II/**1**, apart from having the coumarin ring substituted with a hydrogen atom and the other two hydrogen atoms substituted by fluorine atoms. The model hCA II/2*_ring_ is identical to the crystallographic structure hCA II/**2** apart from having the coumarin ring rotated of 180° thus resembling the orientation of the tail in the hydrogenated derivative **1**. All the model adducts were built with Insight II software (Insight2000, Accelrys, San Diego, CA, USA).

Concerning the ligands, their partial atomic charges were determined using the restrained electrostatic potential (RESP) protocol implemented in the PyRED server [33] through quantum mechanical calculations with the Gaussian16 software (Gaussian, Inc., Wallingford, CT, USA). The total charge for sulfonamide ligands was considered equal to −1 e because they bind the zinc ion in a deprotonated form [34].

The molecular mechanics/generalised Born surface area (MM/GBSA) [35,36] method, implemented in AmberTools18 [37], was used to compute the protein-ligand binding free energies. AMBERff14SB [38] and General AMBER [39] force fields were employed for the proteins and ligands, respectively. For the Zn^2+^ ion, the Van der Waals parameters (σ = 1.271; ε (kcal/mol) = 0.00330286) from the work of Li and Merz [40] were used. In agreement with our previous works [41,42], the charge for the zinc ion was set to +1.5 e. Moreover, to identify important residues for binding, a *per-residue* decomposition of the binding free energy was performed.

According to MM/GBSA method, the binding free energy was estimated as follows:

ΔG_bind_ = G_complex_ − G_protein_ − G_ligand_

where ΔG_bind_ represents the binding free energy and G_complex_, G_protein_, and G_ligand_ are the free energies of complex, protein, and ligand, respectively. In particular:

ΔG_bind_ = ΔE_MM_ + ΔG_sol_ − TΔS

ΔE_MM_= ΔE_elec_ + ΔE_vdW_

ΔG_sol_ = ΔG_GB_ + ΔG_SA_

where ΔG_bind_ is the binding free energy in solution; ΔE_MM_ is the molecular mechanics energy including van der Waals (ΔE_vdW_) and electrostatic (ΔE_elec_) contributions; and ΔG_sol_ is the solvation energy, and is the sum of electrostatic (ΔG_GB_) and nonpolar (ΔG_SA_) interactions. TΔS represents the entropic change due to ligand binding. Our calculations do not include this entropic term, since it is reasonable to exclude it when comparing similar ligands [36,43], in agreement with protocols followed in our previous works [41,44]. ΔG_GB_ is the electrostatic solvation energy and is computed by the Generalized Born method [45], whereas the non-polar contribution is calculated through the Linear Combination of Pairwise Overlaps (LCPO) method [46].

## 3. Results and Discussion

The crystal structures of hCA II in complex with compounds **1** and **2** were determined to resolutions of 1.67 Å and 1.56 Å, respectively.

**Figure 3:**
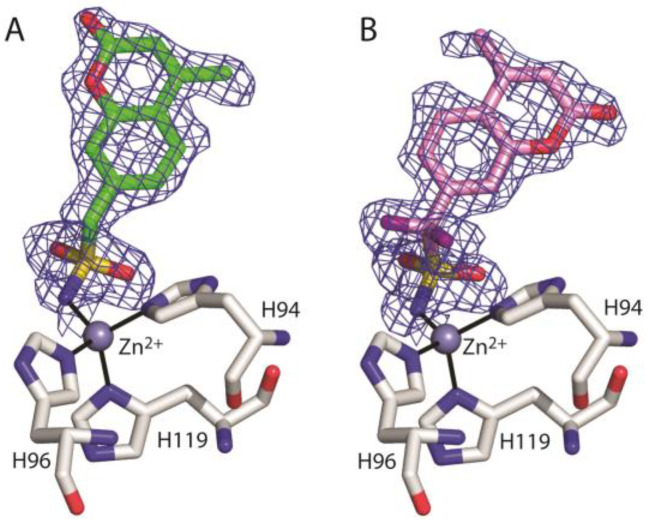
σA-weighted (|2F_o_-F_c_|, ϕ_c_) simulated annealing omit map (contoured at 1.0 σ) relative to the inhibitor molecule in the hCA II/**1** (**A**) and hCA II/**2** (**B**) complexes.

Data collection and refinement statistics for each complex structure are shown in Table 1. In both adducts, inspection of |F_o_-F_c_| and |2F_o_-F_c_| electron density maps (Figure 3) during crystallographic refinement immediately revealed the binding of the inhibitor molecule in the active site. This binding does not generate significant hCA II structural changes; in fact, the r.m.s.d. value calculated by superposition between the Cα atoms of the native enzyme and those of the hCA II/**1** and hCA II/**2** adducts was 0.3 Å.

**Figure 4:**
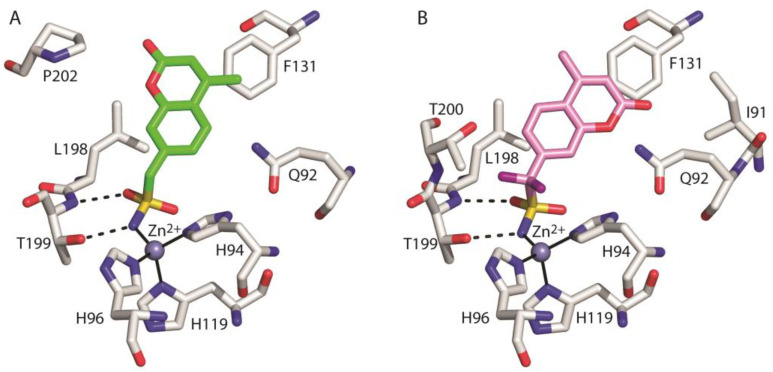
Details of the interactions of compounds **1** (**A**) and **2** (**B**) with the enzyme active site. Residues involved in hydrogen bonds and van der Waals (<4 Å) interactions are shown. Continuous lines indicate zinc ion coordination, whereas dashed lines indicate hydrogen bond distances.

As generally observed for other hCAIs containing the sulfonamide moiety [1], in both adducts the inhibitor binds to the enzyme active site coordinating the catalytic zinc ion through the ionized sulfonamide NH^-^ group and forming further hydrogen bond interactions with Thr199 residue (Figure 4). Interestingly, even if the coumarin moiety is a well-known chemotype for CA inhibition, in the case of compounds **1** and **2**, it does not adopt its typical suicide inhibition mechanism [47,48], due to the predominant effect of the sulfonamide moiety as zinc binding group (ZBG). Instead, in these compounds the coumarin ring contributes to the stabilization of the complex by means of numerous van der Waals interactions with the side chains of several residues delimiting the active site cavity (Figure 4).

**Figure 5:**
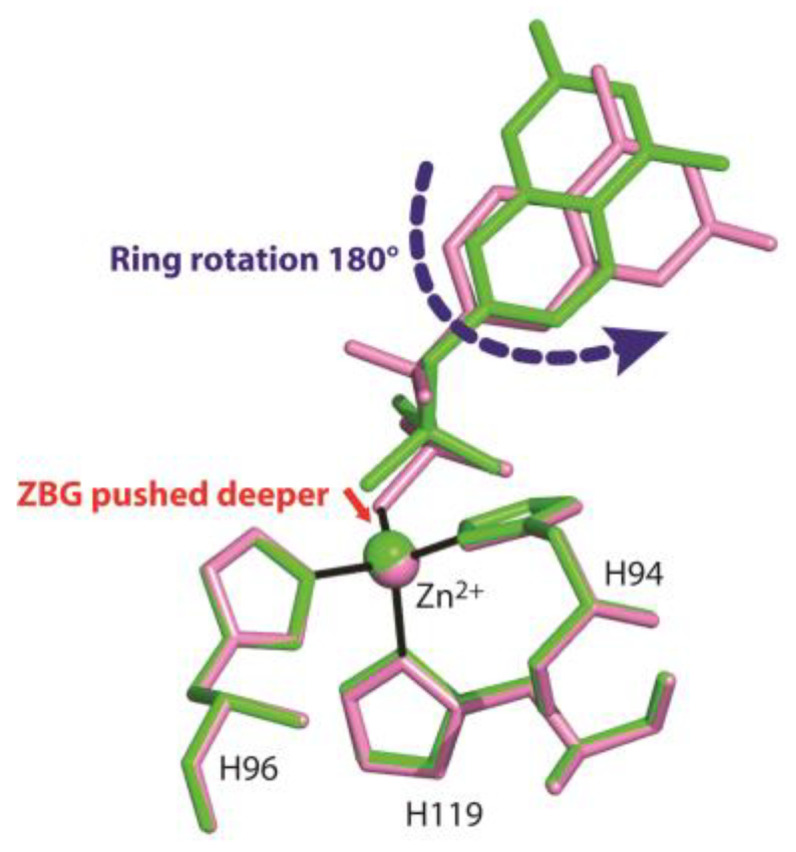
Structural superposition between compounds **1** (green) and **2** (pink) when bound to the hCA II active site.

Figure 5 shows the superposition of the two structures in the region of the active site. Interestingly, even if compounds **1** and **2** differ only by two atoms (two fluorine atoms instead of two hydrogens) (see Figure 2), their arrangement in the enzyme active site is significantly different. In particular, the two coumarin rings present a different orientation and the ZBG in compound **2** is shifted a little bit more deeply inside the catalytic cavity (Figure 5).

Previously reported inhibition studies [23] showed that compound **1** was an hCA II inhibitor three times more potent than compound **2**, suggesting that its binding conformation was energetically favoured with respect to that adopted by the fluorine derivative. Thus, we wondered why the latter did not adopt the same binding mode shown by compound **1**. To answer this question, binding free energy calculations were performed, using the MM/GBSA theoretical method [35,36]. This method allows decomposing the protein-ligand binding free energy on a *per-residue* basis, to identify key protein residues responsible for the inhibitor binding mode. With the aim of evaluating the energetic effects separately due to the different ZBG positions and coumarin ring rotation observed for the two inhibitors, the calculations were carried out on the hCA II/**2** crystallographic adduct and on three other model adducts, hereafter indicated as hCA II/**2_ZBG_**, hCA II/2*_ZBG,_ and hCA II/2*_ring_. **2_ZBG_** and 2*_ZBG_, represent simplified models of the two inhibitors (**2** and **1**) including only the ZBGs. In particular, the model hCA II/**2_ZBG_** corresponds to the crystallographic structure hCA II/**2** with the inhibitor coumarin ring substituted with a hydrogen atom, whereas hCA II/2*_ZBG_ is identical to the hCA II/**1** crystal structure apart from having the ring substituted by a hydrogen atom and the other two hydrogen atoms substituted by fluorine atoms, thus representing a hypothetical model in which the ZBG of the fluorinated derivative **2** would adopt the same binding position observed for the corresponding hydrogenated derivative. Finally, hCA II/2*_ring_ is the hCA II/**2** crystallographic structure with the coumarin ring of **2** rotated 180°, thus corresponding to the orientation of the tail in the hydrogenated derivative **1**.

Firstly, we compared results obtained for hCA II/**2_ZBG_** and hCA II/2*_ZBG_; Table 2 reports protein residues giving a major contribution to the protein-ligand binding energy; the zinc ion contribution is not reported since, as described in literature, it is affected by the overestimation of the electrostatic interactions due to the high positive charge of the Zn^2+^ ion [42,49].

**Figure 6:**
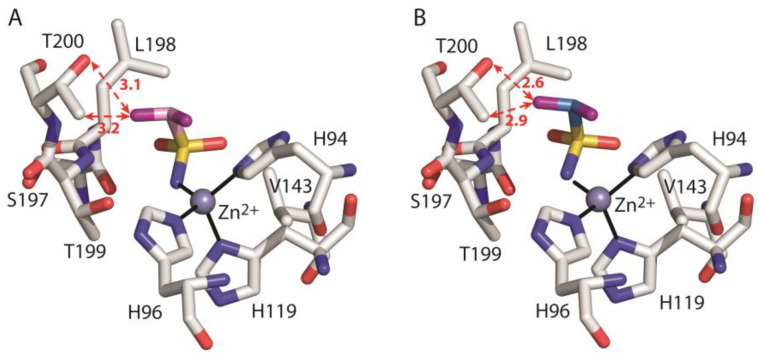
Detail of the active site in the model systems hCA II/**2_ZBG_** (**A**) and hCA II/2*_ZBG_ (**B**). The ligand, the zinc ion with the three coordinating histidines and enzyme residues which give a major contribution to ligand binding are shown. The hydrogen atoms have been omitted for clarity. The distances (in Angstroms) between one of the ligand fluorine atoms and T200 Oγ and Cγ atoms are indicated with red dotted arrows.

Data analysis shows that there are significant energetic differences between the two models mainly due to the unfavourable interaction with residue Thr200 in the case of hCA II/2*_ZBG_ with respect to hCA II**/2_ZBG_** (ΔG_bind_-Thr200 values of 2.238 kcal/mol and −0.512 kcal/mol, respectively). Indeed, in hCA II/2*_ZBG_, the substitution of the short CH bonds (1.1 Å) with the longer CF ones (1.3 Å), leads to a reduction in the distances between one of the inhibitor fluorine atoms and the Thr200 side chain atoms (Figure 6). This distance reduction is likely responsible for the unfavourable contribution of the van der Waals (ΔE_vdW_) term and for the increase in the electrostatic (ΔE_elec_) term, that in hCA II**/2_ZBG_** was already disadvantageous (Table 2). In particular, the increase in electrostatic repulsion could be due to the interaction between inhibitor fluorine and Thr200Oγ atom. Indeed, both atoms have slightly negative partial charges and are at a short distance of only 2.6 Å in hCA II/2*_ZBG_ with respect to 3.1 Å in hCA II/**2_ZBG_** (Figure 6). Thus, according to our calculations, we can hypothesize that the ZBG region of the fluorinated compound **2** assumes a different position with respect to the corresponding hydrogenated derivative **1** to avoid unfavourable steric and electrostatic interactions with Thr200 at the bottom of the active site.

Subsequently, calculations were carried out on the crystallographic adduct hCA II/**2** and the model adduct hCA II/2*_ring_. The obtained data are listed in Table 3 and show that both ligand conformations (**2** and 2*_ring_) are involved in stabilizing interactions with residues Ile91, Gln92, and Val121; however, in the case of 2*_ring_, these interactions are weaker with respect to **2**. Moreover, 2*_ring_ is affected by strong destabilizing interactions with Phe131 and Pro202 (*per residue* ΔG_bind_ equal to 34.388 and 11.394 kcal/mol, respectively) mainly due to van der Waals contributions (see Table 3). 

**Figure 7:**
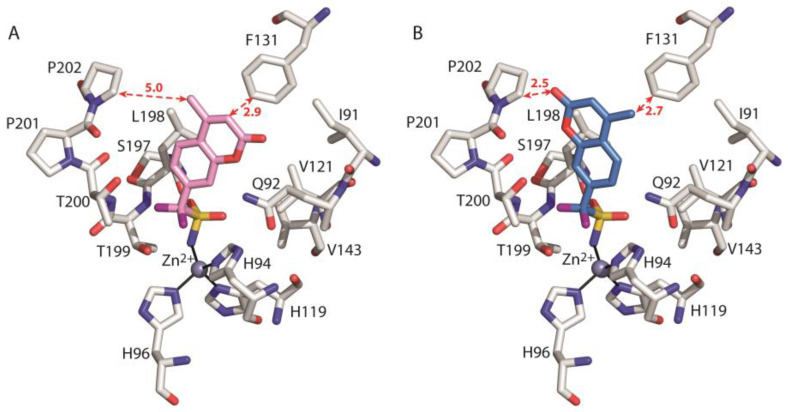
Detail of the active site in the crystallographic adduct hCA II/**2** (**A**) and the model hCA II/2*_ring_ (**B**). The ligand, the zinc ion, the three coordinating histidines and enzyme residues which give a major contribution to ligand binding are shown. The hydrogen atoms have been omitted for clarity. The distances (in Angstroms) between the ligand coumarin ring and the side chains of F131 and P202 are indicated with red dotted arrows.

Indeed, as shown in Figure 7, in the case of the model adduct hCA II/2*_ring_, the coumarin ring and its methyl substituent are too close to the side chains of Phe131 and Pro202, respectively, leading to steric repulsion. These results indicate that the fluorinated compound **2** would experience significant destabilizing interactions with Pro202 and Phe131 if its coumarin ring would adopt the same orientation as the corresponding hydrogenated derivative **1**.

Overall, our energetic calculations show that in aliphatic sulfonamides with general formula R-CH_2_-SO_2_NH_2_, the replacement of two aliphatic hydrogen atoms with two bulky and electronegative fluorine atoms can have important effects on the binding conformation not only locally at the bottom of the active site, but even remotely at the entrance of the catalytic cavity, thanks to a delicate balance of electrostatic and steric effects involving enzyme residues.

In conclusion, the combined crystallographic and computational study reported here describes in detail the structural features and the energetic factors involved in the interaction of hCA II with a promising class of aliphatic sulfonamides incorporating coumarin rings. In agreement with previous studies [1,42,50], it was highlighted that residue Thr200 at the bottom of the enzyme catalytic cavity and hydrophobic residues Phe131 and Pro202 at its entrance, play key roles in modulating the interaction with inhibitor molecules. Moreover, our studies also highlight that in aliphatic sulphonamide of the R-CH_2_-SO_2_NH_2_ type, the introduction of substituents on the aliphatic carbon atom can be used to modulate the affinity of the inhibitor against a specific CA isoform. In this respect, the present study represents a step forward in the rational design of novel isozyme-specific inhibitor molecules with improved features.

## Figures and Tables

**Table 1:** Data collection and refinement statistics for hCA II/**1** and hCA II/**2** complexes.

	hCA II/1	hCA II/2
*Crystal parameters*		
Space group	P2_1_	P2_1_
a (Å)	42.3	42.3
b (Å)	41.5	41.5
c (Å)	72.2	72.1
β (°)	104.4	104.4
*Data collection statistics*		
Resolution (Å)	25.4–1.67 (1.70–1.67)	24.6–1.56 (1.59–1.56)
Temperature (K)	100	100
Total reflections	104,361	141,168
Unique reflections	26,790	33,655
Completeness (%)	94.0 (81.3)	96.4 (71.9)
<I>/<σ(I)>	15.2 (2.1)	15.9 (2.5)
Redundancy (%)	3.9 (2.9)	4.2 (2.3)
R_merge_ ^a^	0.073 (0.591)	0.070 (0.359)
R_meas_ ^b^	0.082 (0.703)	0.077 (0.448)
R_pim_ ^c^	0.038 (0.374)	0.032 (0.261)
CC1/2 ^d^	0.998 (0.622)	0.998 (0.801)
*Refinement statistics*		
Resolution (Å)	25.4–1.67	24.6–1.56
R_work_ ^e^ (%)	17.5	17.7
R_free_ ^e^ (%)	21.8	20.1
r.m.s.d. from ideal geometry:		
Bond lengths (Å)	0.009	0.009
Bond angles (°)	1.6	1.6
Number of protein atoms	2076	2072
Number of inhibitor atoms	17	19
Number of water molecules	219	238
Average B factor (Å^2^)		
All atoms	15.5	12.7
Protein atoms	14.7	11.7
Inhibitor atoms	18.1	16.9
Water molecules	23.2	20.9

^a^ R_merge_ = ∑_hkl_∑_i_|I_i_(hkl)-<I(hkl)>|/∑_hkl_∑_i_I_i_(hkl); ^b^ R_meas_ = ∑_hkl_{n(hkl)/[n(hkl)-1]}^1/2^∑_i_|I_i_(hkl)-<I(hkl)>|/∑_hkl_∑_i_I_i_(hkl); ^c^ R_pim_ = ∑_hkl_{1/[n(hkl)-1]}^1/2^∑_i_|I_i_(hkl)-<I(hkl)>|/∑_hkl_∑_i_I_i_(hkl), where I_i_(hkl) is the intensity of an observation and <I(hkl)> is the mean value for its unique reflection; summations are over all “n” reflections. ^d^ CC1/2 = [∑_i_(a_i_-<a>)/∑_i_(b_i_-<b>)]/[∑_i_(a_i_-<a>)^2^ ∑_i_(b_i_-<b>)^2^]^1/2^; where a_i_ and b_i_ are the intensities of unique reflections merged across the observations randomly assigned to subsets A and B, respectively, and <a> and <b> are their averages. ^e^ R_work_ = ∑_h_||F_o_(h)|-|F_c_(h)||/∑_h_|Fo(h)|, where F_o_ and F_c_ are the observed and calculated structure-factor amplitudes, respectively. R_free_ was calculated with 2.4% (hCA II/**1**) or 2.6% (hCA II/**2**) of the data excluded from the refinement. Values in parentheses are referred to the highest resolution shell.

**Table 2:** *Per-residue* decomposition of the binding free energy (kcal/mol) computed by the MM/GBSA method for the model complexes hCA II/**2_ZBG_** and hCA II/2*_ZBG_. Details of the ΔG_bind_-Thr200 energy terms (kcal/mol) are shown in light grey lines.

		hCA II/2_ZBG_	hCA II/2*_ZBG_
ΔG_bind_-Val143		−1.234	−0.988
ΔG_bind_-Ser197		−0.968	−1.130
ΔG_bind_-Leu198		−5.170	−5.644
ΔG_bind_-Thr199		−1.890	−0.715
ΔG_bind_-Thr200		−0.512	2.238
	ΔE_vdW_	−0.003	2.310
	ΔE_elec_	2.510	3.008
	ΔG_GB_	−2.358	−2.259
	ΔG_SA_	−0.661	−0.821

ΔE_vdW_: van der Waals contribution; ΔE_elec_: electrostatic contribution; ΔG_GB_: generalised-Born solvation contribution; ΔG_SA_: non-polar solvation contribution.

**Table 3:** *Per-residue* decomposition of the binding free energy (kcal/mol) computed by the MM/GBSA method for the crystallographic adduct hCA II/**2** and the model adduct hCA II/2*_ring_. Details of ΔG_bind_-Phe131 and ΔG_bind_-Pro202 energy terms (kcal/mol) are shown in light grey lines.

		hCA II/2	hCA II/2*_ring_
ΔG_bind_-Ile91		−1.158	−0.354
ΔG_bind_-Gln92		−2.726	−1.271
ΔG_bind_-Val121		−2.332	−1.622
ΔG_bind_-Phe131		−1.117	34.388
	ΔE_vdW_	−0.131	35.858
	ΔE_elec_	0.169	−0.240
	ΔG_GB_	0.128	0.063
	ΔG_SA_	−1.283	−1.293
ΔG_bind_-Val143		−1.266	−1.254
ΔG_bind_-Ser197		−0.944	−0.939
ΔG_bind_-Leu198		−7.033	−6.781
ΔG_bind_-Thr199		−2.203	−2.146
ΔG_bind_-Thr200		−1.426	−2.008
ΔG_bind_-Pro201		−0.122	−0.679
ΔG_bind_-Pro202		−0.766	11.394
	ΔE_vdW_	−0.364	12.442
	ΔE_elec_	−0.340	−1.797
	ΔG_GB_	0.277	1.336
	ΔG_SA_	−0.340	−0.586

ΔE_vdW_: van der Waals contribution; ΔE_elec_: electrostatic contribution; ΔG_GB_: eneralized-Born solvation contribution; ΔG_SA_: non-polar solvation contribution.

## Data Availability

Crystallographic data are available in the RCSB PDB database under the codes: 8C0Q, 8C0R.

## References

[1] Alterio V., Di Fiore A., D’Ambrosio K., Supuran C.T., De Simone G. (2012). Multiple Binding Modes of Inhibitors to Carbonic Anhydrases: How to Design Specific Drugs Targeting 15 Different Isoforms?. Chem. Rev..

[2] Langella E., Di Fiore A., Alterio V., Monti S.M., De Simone G., D’Ambrosio K. (2022). α-CAs from Photosynthetic Organisms. Int. J. Mol. Sci..

[3] Alterio V., Langella E., Buonanno M., Esposito D., Nocentini A., Berrino E., Bua S., Polentarutti M., Supuran C.T., Monti S.M. (2021). Zeta-Carbonic Anhydrases Show CS_2_ Hydrolase Activity: A New Metabolic Carbon Acquisition Pathway in Diatoms?. Comput. Struct. Biotechnol. J..

[4] Jensen E.L., Clement R., Kosta A., Maberly S.C., Gontero B. (2019). A New Widespread Subclass of Carbonic Anhydrase in Marine Phytoplankton. ISME J..

[5] Kikutani S., Nakajima K., Nagasato C., Tsuji Y., Miyatake A., Matsuda Y. (2016). Thylakoid Luminal θ-Carbonic Anhydrase Critical for Growth and Photosynthesis in the Marine Diatom *Phaeodactylum Tricornutum*. Proc. Natl. Acad. Sci. USA.

[6] Ferry J.G. (2010). The γ-Class of Carbonic Anhydrases. Biochim. Biophys. Acta (BBA)-Proteins Proteom..

[7] Zimmerman S., Ferry J. (2008). The β- and γ-Classes of Carbonic Anhydrase. Curr. Pharm. Des..

[8] De Simone G., Di Fiore A., Capasso C., Supuran C.T. (2015). The Zinc Coordination Pattern in the η-Carbonic Anhydrase from *Plasmodium Falciparum* Is Different from All Other Carbonic Anhydrase Genetic Families. Bioorg. Med. Chem. Lett..

[9] Alterio V., Langella E., Viparelli F., Vullo D., Ascione G., Dathan N.A., Morel F.M.M., Supuran C.T., De Simone G., Monti S.M. (2012). Structural and Inhibition Insights into Carbonic Anhydrase CDCA1 from the Marine Diatom *Thalassiosira Weissflogii*. Biochimie.

[10] Hirakawa Y., Senda M., Fukuda K., Yu H.Y., Ishida M., Taira M., Kinbara K., Senda T. (2021). Characterization of a Novel Type of Carbonic Anhydrase That Acts without Metal Cofactors. BMC Biol..

[11] Vullo D., Del Prete S., Di Fonzo P., Carginale V., Donald W.A., Supuran C.T., Capasso C., Mcphee D.J., Muñoz-Torrero D. (2017). Comparison of the Sulfonamide Inhibition Profiles of the β- and γ-Carbonic Anhydrases from the Pathogenic Bacterium *Burkholderia pseudomallei*. Molecules.

[12] Truppo E., Supuran C.T., Sandomenico A., Vullo D., Innocenti A., Di Fiore A., Alterio V., De Simone G., Monti S.M. (2012). Carbonic Anhydrase VII Is S-Glutathionylated without Loss of Catalytic Activity and Affinity for Sulfonamide Inhibitors. Bioorg. Med. Chem. Lett..

[13] Langella E., Buonanno M., Vullo D., Dathan N., Leone M., Supuran C.T., De Simone G., Monti S.M. (2018). Biochemical, Biophysical and Molecular Dynamics Studies on the Proteoglycan-like Domain of Carbonic Anhydrase IX. Cell. Mol. Life Sci..

[14] Supuran C.T. (2008). Carbonic Anhydrases: Novel Therapeutic Applications for Inhibitors and Activators. Nat. Rev. Drug Discov..

[15] Supuran C.T., De Simone G. (2015). Carbonic Anhydrases as Biocatalysts.

[16] De Simone G., Alterio V., Supuran C.T. (2013). Exploiting the Hydrophobic and Hydrophilic Binding Sites for Designing Carbonic Anhydrase Inhibitors. Expert Opin. Drug Discov..

[17] Maren T.H., Conroy C.W. (1993). A New Class of Carbonic Anhydrase Inhibitor. J. Biol. Chem..

[18] Remko M., Von Der Lieth C.W. (2004). Theoretical Study of Gas-Phase Acidity, PKa, Lipophilicity, and Solubility of Some Biologically Active Sulfonamides. Bioorg. Med. Chem..

[19] Dvořanová J., Kugler M., Holub J., Šícha V., Das V., Nekvinda J., El Anwar S., Havránek M., Pospíšilová K., Fábry M. (2020). Sulfonamido Carboranes as Highly Selective Inhibitors of Cancer-Specific Carbonic Anhydrase IX. Eur. J. Med. Chem..

[20] Grüner B., Kugler M., El Anwar S., Holub J., Nekvinda J., Bavol D., Růžičková Z., Pospíšilová K., Fábry M., Král V. (2021). Cobalt Bis(Dicarbollide) Alkylsulfonamides: Potent and Highly Selective Inhibitors of Tumor Specific Carbonic Anhydrase IX. ChemPlusChem.

[21] De Simone G., Di Fiore A., Menchise V., Pedone C., Antel J., Casini A., Scozzafava A., Wurl M., Supuran C.T. (2005). Carbonic Anhydrase Inhibitors. Zonisamide Is an Effective Inhibitor of the Cytosolic Isozyme II and Mitochondrial Isozyme V: Solution and X-Ray Crystallographic Studies. Bioorg. Med. Chem. Lett..

[22] Temperini C., Cecchi A., Boyle N.A., Scozzafava A., Cabeza J.E., Wentworth P., Blackburn G.M., Supuran C.T. (2008). Carbonic Anhydrase Inhibitors. Interaction of 2-N,N-Dimethylamino-1,3,4-Thiadiazole-5-Methanesulfonamide with 12 Mammalian Isoforms: Kinetic and X-Ray Crystallographic Studies. Bioorg. Med. Chem. Lett..

[23] Cecchi A., Taylor S.D., Liu Y., Hill B., Vullo D., Scozzafava A., Supuran C.T. (2005). Carbonic Anhydrase Inhibitors: Inhibition of the Human Isozymes I, II, VA, and IX with a Library of Substituted Difluoromethanesulfonamides. Bioorg. Med. Chem. Lett..

[24] Hill B., Liu Y., Taylor S.D. (2004). Synthesis of α-Fluorosulfonamides by Electrophilic Fluorination. Org. Lett..

[25] De Simone G., Angeli A., Bozdag M., Supuran C.T., Winum J.Y.J.-Y., Monti S.M., Alterio V. (2018). Inhibition of Carbonic Anhydrases by a Substrate Analog: Benzyl Carbamate Directly Coordinates the Catalytic Zinc Ion Mimicking Bicarbonate Binding. Chem. Commun..

[26] Alterio V., De Simone G., Monti S.M., Scozzafava A., Supuran C.T. (2007). Carbonic Anhydrase Inhibitors: Inhibition of Human, Bacterial, and Archaeal Isozymes with Benzene-1,3-Disulfonamides-Solution and Crystallographic Studies. Bioorg. Med. Chem. Lett..

[27] Otwinowski Z., Minor W. (1997). Processing of X-Ray Diffraction Data Collected in Oscillation Mode. Methods Enzymol..

[28] Alterio V., Tanc M., Ivanova J., Zalubovskis R., Vozny I., Monti S.M., Di Fiore A., De Simone G., Supuran C.T. (2015). X-Ray Crystallographic and Kinetic Investigations of 6-Sulfamoyl-Saccharin as a Carbonic Anhydrase Inhibitor. Org. Biomol. Chem..

[29] Jones T.A., Zou J.-Y., Cowan S.W., Kjeldgaard M. (1991). Improved Methods for Building Protein Models in Electron Density Maps and the Location of Errors in These Models. Acta Crystallogr. Sect. A Found. Crystallogr..

[30] Brunger A.T. (2007). Version 1.2 of the Crystallography and NMR System. Nat. Protoc..

[31] Brünger A.T., Adams P.D., Clore G.M., Delano W.L., Gros P., Grossekunstleve R.W., Jiang J.S., Kuszewski J., Nilges M., Pannu N.S. (1998). Crystallography & NMR System: A New Software Suite for Macromolecular Structure Determination. Acta Crystallogr. Sect. D Biol. Crystallogr..

[32] Groom C.R., Bruno I.J., Lightfoot M.P., Ward S.C. (2016). The Cambridge Structural Database. Acta Crystallogr. Sect. B: Struct. Sci. Cryst. Eng. Mater..

[33] Vanquelef E., Simon S., Marquant G., Garcia E., Klimerak G., Delepine J.C., Cieplak P., Dupradeau F.Y.R.E.D. (2011). Server: A Web Service for Deriving RESP and ESP Charges and Building Force Field Libraries for New Molecules and Molecular Fragments. Nucleic Acids Res..

[34] Taylor P.W., King R.W., Burgen A.S.V. (1970). Influence of PH on the Kinetics of Complex Formation between Aromatic Sulfonamides and Human Carbonic Anhydrase. Biochemistry.

[35] Tsui V., Case D.A. (2000). Theory and Applications of the Generalized Born Solvation Model in Macromolecular Simulations. Biopolymers.

[36] Kollman P.A., Massova I., Reyes C., Kuhn B., Huo S., Chong L., Lee M., Lee T., Duan Y., Wang W. (2000). Calculating Structures and Free Energies of Complex Molecules: Combining Molecular Mechanics and Continuum Models. Acc. Chem. Res..

[37] Case D.A., Ben-Shalom I.Y., Brozell S.R., Cerutti D.S., Cheatham T.E.I., Cruzeiro V.W.D., Darden T., Duke R.E., Ghoreishi D., Gilson M.K. (2018). Amber 18.

[38] Maier J.A., Martinez C., Kasavajhala K., Wickstrom L., Hauser K.E., Simmerling C. (2015). Ff14SB: Improving the Accuracy of Protein Side Chain and Backbone Parameters from Ff99SB. J. Chem. Theory Comput..

[39] Wang J., Wolf R.M., Caldwell J.W., Kollman P.A., Case D.A. (2004). Development and Testing of a General Amber Force Field. J. Comput. Chem..

[40] Li P., Merz K.M. (2014). Taking into Account the Ion-Induced Dipole Interaction in the Nonbonded Model of Ions. J. Chem. Theory Comput..

[41] De Simone G., Langella E., Esposito D., Supuran C.T., Monti S.M., Winum J.-Y., Alterio V. (2017). Insights into the Binding Mode of Sulphamates and Sulphamides to hCA II: Crystallographic Studies and Binding Free Energy Calculations. J. Enzyme Inhib. Med. Chem..

[42] Langella E., Alterio V., D’Ambrosio K., Cadoni R., Winum J.-Y., Supuran C.T., Monti S.M., De Simone G., Di Fiore A. (2019). Exploring Benzoxaborole Derivatives as Carbonic Anhydrase Inhibitors: A Structural and Computational Analysis Reveals Their Conformational Variability as a Tool to Increase Enzyme Selectivity. J. Enzyme Inhib. Med. Chem..

[43] Wang J., Morin P., Wang W., Kollman P.A. (2001). Use of MM-PBSA in Reproducing the Binding Free Energies to HIV-1 RT of TIBO Derivatives and Predicting the Binding Mode to HIV-1 RT of Efavirenz by Docking and MM-PBSA. J. Am. Chem. Soc..

[44] Autiero I., Saviano M., Langella E. (2015). Conformational Studies of Chiral D-Lys-PNA and Achiral PNA System in Binding with DNA or RNA through a Molecular Dynamics Approach. Eur. J. Med. Chem..

[45] Onufriev A., Bashford D., Case D.A. (2004). Exploring Protein Native States and Large-Scale Conformational Changes with a Modified Generalized Born Model. Proteins Struct. Funct. Genet..

[46] Weiser J., Shenkin P.S., Still W.C. (1999). Approximate Solvent-Accessible Surface Areas from Tetrahedrally Directed Neighbor Densities. Biopolymers.

[47] Maresca A., Temperini C., Vu H., Pham N.B., Poulsen S.A., Scozzafava A., Quinn R.J., Supuran C.T. (2009). Non-Zinc Mediated Inhibition of Carbonic Anhydrases: Coumarins Are a New Class of Suicide Inhibitors. J. Am. Chem. Soc..

[48] Maresca A., Temperini C., Pochet L., Masereel B., Scozzafava A., Supuran C.T. (2010). Deciphering the Mechanism of Carbonic Anhydrase Inhibition with Coumarins and Thiocoumarins. J. Med. Chem..

[49] Hou T., Wang J., Li Y., Wang W. (2011). Assessing the Performance of the MM/PBSA and MM/GBSA Methods: I. The Accuracy of Binding Free Energy Calculations Based on Molecular Dynamics Simulations. J. Chem. Inf. Model..

[50] Menchise V., De Simone G., Alterio V., Di Fiore A., Pedone C., Scozzafava A., Supuran C.T. (2005). Carbonic Anhydrase Inhibitors: Stacking with Phe131 Determines Active Site Binding Region of Inhibitors as Exemplified by the X-Ray Crystal Structure of a Membrane-Impermeant Antitumor Sulfonamide Complexed with Isozyme II. J. Med. Chem..

